# Formononetin Exerts Neuroprotection in Parkinson’s Disease via the Activation of the Nrf2 Signaling Pathway

**DOI:** 10.3390/molecules29225364

**Published:** 2024-11-14

**Authors:** Xiaotong Wang, Nianxin Kang, Ying Liu, Guojie Xu

**Affiliations:** School of Life Sciences, Beijing University of Chinese Medicine, Beijing 102488, China; wxt970907@126.com (X.W.); knx_0423@163.com (N.K.)

**Keywords:** Parkinson’s disease, formononetin, Nrf2, mitochondrion

## Abstract

Parkinson’s disease (PD) is a prevalent neurodegenerative disease for which no effective treatment currently exists. In this study, we identified formononetin (FMN), a neuroprotective component found in herbal medicines such as *Astragalus membranaceus* and *Glycyrrhiza uralensis*, as a potential agent targeting multiple pathways involved in PD. To investigate the anti-PD effects of FMN, we employed *Caenorhabditis elegans* (*C. elegans*) PD models, specifically the transgenic strain NL5901 and the MPP(+)-induced strain BZ555, to investigate the effects of FMN on the key pathological features of PD, including dyskinesia, dopamine neuron damage, and reactive oxygen species (ROS) accumulation. The MPP(+)-induced SH-SY5Y cell PD model was utilized to evaluate the effects of FMN on cell viability, ROS accumulation, and mitochondrial dysfunction. The signaling pathway induced by FMN was analyzed using transcriptomic techniques and subsequently validated in vitro. Our results indicate that FMN significantly reduced ROS accumulation and improved both dopaminergic neuron vitality and dyskinesia in the *C. elegans* PD models. In the cell PD model, FMN significantly reduced ROS accumulation and enhanced mitochondrial membrane potential (MMP) and cell viability. A transcriptomic analysis suggested that the effects of FMN are associated with Nrf2 activation. Furthermore, ML385, a specific Nrf2 inhibitor, blocked the beneficial effects of FMN in vitro, indicating that FMN ameliorates dyskinesia and protects dopaminergic neurons through Nrf2 signaling pathway activation. In addition, the effects of FMN on ameliorating dyskinesia and protecting dopamine neurons were comparable to those of the Nrf2 agonist of sulforaphane (SFN) in vivo. The results of this study confirm that FMN exerts significant anti-PD effects primarily through the Nrf2 signaling pathway. These findings provide crucial insights for the development of anti-PD therapies.

## 1. Introduction

Parkinson’s disease (PD) is the second most prevalent neurodegenerative disease, characterized by the loss of dopaminergic neurons, the accumulation of α-synuclein proteins, and mitochondrial dysfunction, and it clinically manifests as movement disorders such as resting tremor, bradykinesia, postural instability, and rigidity in various parts of the body [1,2,3,4,5]. Data from the Global Burden of Disease study show that PD has the fastest growth rate among neurodegenerative diseases. Between 1990 and 2015, the number of PD cases increased by 6%, reaching 1.18 million cases worldwide [6]. By 2040, the global prevalence of PD is expected to double [7], imposing a significant and evident burden on society and individuals. Current treatment options for PD primarily focus on symptom alleviation, such as the use of levodopa, MAO inhibitors, and dopamine pathway agonists [8,9,10]. However, due to the complex etiology of PD, none of the currently available therapies are truly effective [11,12]. Therefore, there remains an urgent need to find therapeutic agents for PD management.

**Figure 1:**
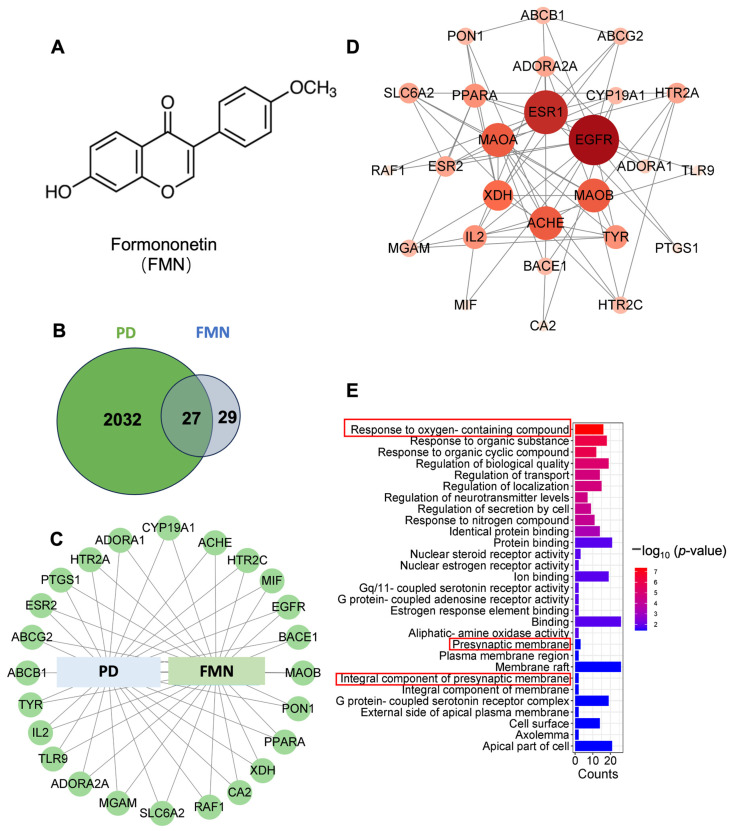
Bioinformatics-based target prediction suggests a potential role for FMN in alleviating Parkinson’s disease (PD). (**A**) An illustration of the chemical structure of FMN. (**B**) A Venn diagram illustrating the overlap between PD-related proteins and FMN targets, created using VENNY (version 2.1, https://bioinfogp.cnb.csic.es/tools/venny/index.html, accessed on 15 March 2024). (**C**) An interactive network visualization of PD-related proteins and FMN targets. (**D**) A Protein–Protein Interaction (PPI) network of shared targets, revealing complex interconnections and potential pathways influenced by FMN in PD. The PPI network was constructed using the String database (version 12.0, http://string-db.org/, accessed on 15 March 2024), identifying 24 nodes and 50 edges. Differently colored nodes represent hub genes. (**E**) A Gene Ontology (GO) enrichment analysis of FMN’s potential targets in PD, highlighting the presynaptic membrane receptor response to oxygen-containing signals in red. The column color indicates significance, and its length reflects the number of genes enriched in the function.

Formononetin (FMN), an isoflavonoid with a benzopyranone core structure (Figure 1A), is an important bioactive compound in herbal medicines (such as *Astragalus membranaceus* and *Glycyrrhiza uralensis*). It exhibits multiple pharmacological activities, including anti-cancer, anti-apoptotic, anti-inflammatory, and antioxidant properties [13,14,15]. Research has shown that FMN can significantly reduce levels of cholesterol, triglycerides, and low-density lipoprotein cholesterol while increasing high-density lipoprotein cholesterol levels, thereby regulating lipid metabolism and exerting anti-atherosclerotic effects [16]. It has also been shown to mitigate oxidative stress and apoptosis in brain tissue following ischemic injury, contributing to recovery from stroke-induced neurodegeneration [17]. The results of studies on neurological diseases have shown that FMN may ameliorate Alzheimer’s disease (AD) and cerebral ischemic stroke by exerting neuroprotective effects, such as inhibiting ROS formation and reducing inflammation [18]. However, the effects of FMN on PD remain unclear.

In this study, we aimed to explore the role of FMN in PD using *Caenorhabditis elegans* (*C. elegans*) PD models (namely NL5901 and BZ555 strains) and MPP(+)-induced cellular PD models [19,20,21,22,23]. Our findings suggest that FMN may ameliorate dyskinesia and protect dopaminergic neurons via the activation of the Nrf2 signaling pathway. The findings of this study could contribute valuable insights for the discovery of novel anti-PD drugs.

## 2. Results

### 2.1. Bioinformatic Target Prediction Revealed a Potential Role of FMN in Alleviating PD

The results of previous studies have demonstrated the neuroprotective effects of FMN [17,24,25,26], though its effects on PD remain unclear. To explore the predicted effects and targets of FMN in PD, we screened 2059 PD-related targets using the GeneCards and OMIM databases. Through a VENNY2.1 analysis, we identified 56 FMN targets, 27 of which were common to both FMN and PD (Figure 1B,C). A Protein–Protein Interaction (PPI) network analysis revealed interactions among these 27 common targets (Figure 1D and Table S1). The size of the node and the depth of the color are positively correlated with the degree value. A Gene Ontology (GO) enrichment analysis identified 157 Biological Processes (BPs), 10 Cellular Components (CCs), and 22 Molecular Functions (MFs) associated with these targets. The most enriched terms suggest that FMN-PD interactions may result in presynaptic membrane receptor responses to oxygen-containing signals (Figure 1E).

### 2.2. FMN Demonstrated Neuroprotection Effects in C. elegans PD Models

#### 2.2.1. FMN Reduced Dopaminergic Neuron Damage and Alleviated Dyskinesia in the *C. elegans* PD Model

**Figure 2:**
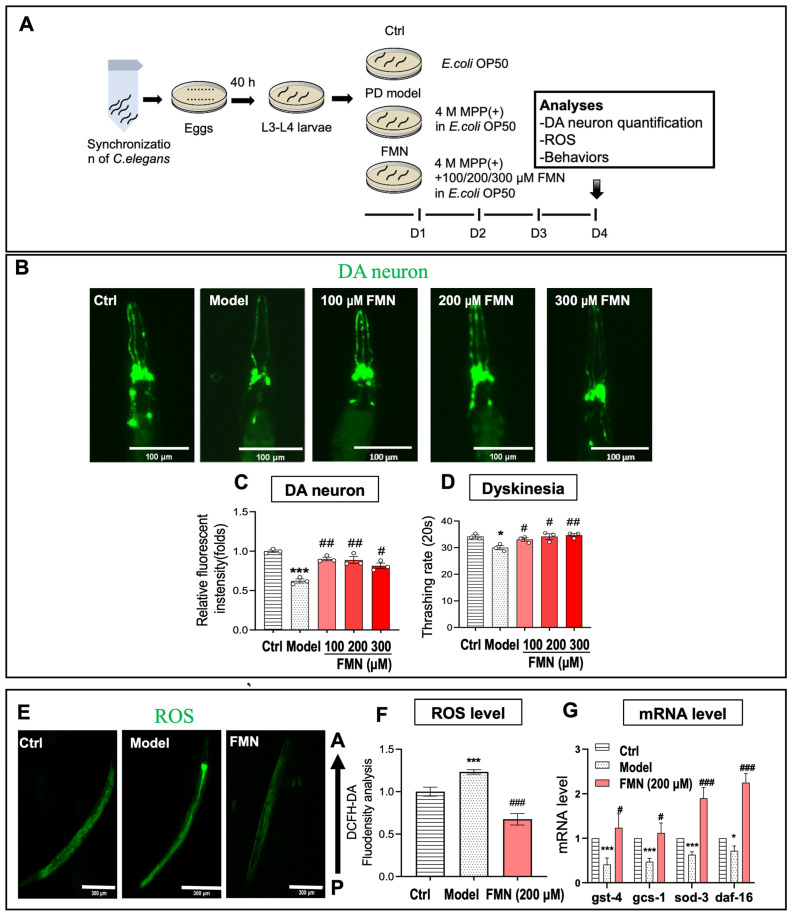
FMN alleviated the key pathological features of PD in the MPP(+)-induced *C. elegans* model. (**A**) Experimental procedure: *C. elegans* at L4 were treated with 4 mM MPP(+) (MPP) or 100 to 300 μM FMN (MPP + FMN) for 4 days at 20 °C, with an untreated group serving as the control (Ctrl). (**B**–**D**) The effects of FMN on dopamine neuron impairment and motor function in MPP(+)-induced *C. elegans* BZ555. (**B**) Representative fluorescent images illustrating the effect of FMN on GFP-labeled dopamine neurons (green) in the head region. Scale bar = 100 μm *(n* = 15; *N* = 3). (**C**) The quantification of fluorescence in dopamine neurons. (**D**) Motor function was assessed through a thrashing rate analysis (*n* = 15; *N* = 3). (**E**–**G**) The effects of 200 μM FMN on ROS and the expression of ROS-related genes in MPP(+)-induced *C. elegans* N2, shown by (**E**) representative fluorescence images and (**F**) the quantification of ROS fluorescence (green). Scale bar = 300 μm (*n* = 15; *N* = 3). (**G**) The expression levels of ROS-related genes quantified by qRT-PCR (*n* = 6; *N* = 3), where *N* = the number of independent experiments and *n* = the number of nematodes in each independent experiment. An asterisk (*) indicates significant differences between the Model and Ctrl groups. A hash mark (#) indicates significant differences between the FMN and Model groups. * *p* < 0.05, *** *p* < 0.001, # *p* < 0.05, ## *p* < 0.01, and ### *p* < 0.001.

PD is characterized by the degeneration of dopaminergic neurons, the aggregation of α-synuclein, and motor dysfunction [2,4]. In this study, we used MPP(+) to induce dopaminergic neuron degeneration in *C. elegans* strain BZ555, which expresses Green Fluorescent Protein (GFP) in dopaminergic neurons. Our findings indicate that MPP(+) treatment significantly reduced the number of GFP-positive dopaminergic neurons. FMN at 100 and 200 μM provided comparable neuroprotection, while FMN at 300 μM showed diminished protective effects, likely due to high-concentration toxicity. These results suggest that FMN effectively mitigates dopaminergic neuron degeneration (Figure 2A–C).

The thrashing rate was used to assess the motility of *C. elegans* [27,28]. To further investigate whether FMN also ameliorates motor dysfunction, we analyzed the thrashing rate of the MPP(+)-induced *C. elegans* strain BZ555. As a result, MPP(+) significantly decreased the thrashing rate of *C. elegans*, while FMN at various concentrations significantly increased the thrashing rate of *C. elegans*, indicating that FMN ameliorates dyskinesia in the MPP(+)-induced *C. elegans* PD model (Figure 2D).

In conclusion, our results demonstrate that FMN could ameliorate motor dysfunction accompanied by typical PD characteristics, including dopaminergic neuron reduction in the *C. elegans* PD model.

#### 2.2.2. FMN Had the Potential to Alleviate Oxidative Stress in the *C. elegans* PD Model

Oxidative stress is closely associated with the progression of PD [29,30,31]. Excessive ROS can cause significant cellular damage under oxidative stress conditions. The results of previous studies have demonstrated that FMN is an effective scavenger of free radicals, indicating its substantial antioxidant capacity [32,33,34]. In this study, we eliminated the ability of FMN to reduce excessive ROS generation in the MPP(+)-induced *C. elegans* PD model, utilizing a fluorescent dihydroethidium (DHE) probe to evaluate ROS levels. The results demonstrate that MPP(+) treatment significantly increased DHE intensity compared to the control group; in comparison, FMN treatment reduced DHE intensity (Figure 2E,F). These findings suggest that FMN could reduce ROS generation and exert antioxidant effects in the MPP(+)-induced *C. elegans* model.

Key antioxidant genes, including gst-4, gcs-1, sod-3, and daf-16, have been identified as important genes in *C. elegans* [35,36,37], and the reduction in ROS may be linked to their expression. To explore whether FMN modulates antioxidant gene expression, we measured the mRNA levels of these genes using real-time fluorescence quantitative PCR (RT-qPCR). As shown in Figure 2G, MPP(+) significantly reduced the expression of gst-4, gcs-1, sod-3, and daf-16; in comparison, FMN treatment notably increased their expression.

These results indicate that FMN could mitigate oxidative stress damage induced by ROS by enhancing the expression of antioxidant genes.

### 2.3. FMN Demonstrated Neuroprotective Effects in the SH-SY5Y Cell Model of PD

SH-SY5Y cells treated with MPP(+) have been used as a cellular model of PD [38,39]. To validate the neuroprotective effects of FMN, we used this model to examine whether FMN could ameliorate oxidative stress and enhance cell viability.

**Figure 3:**
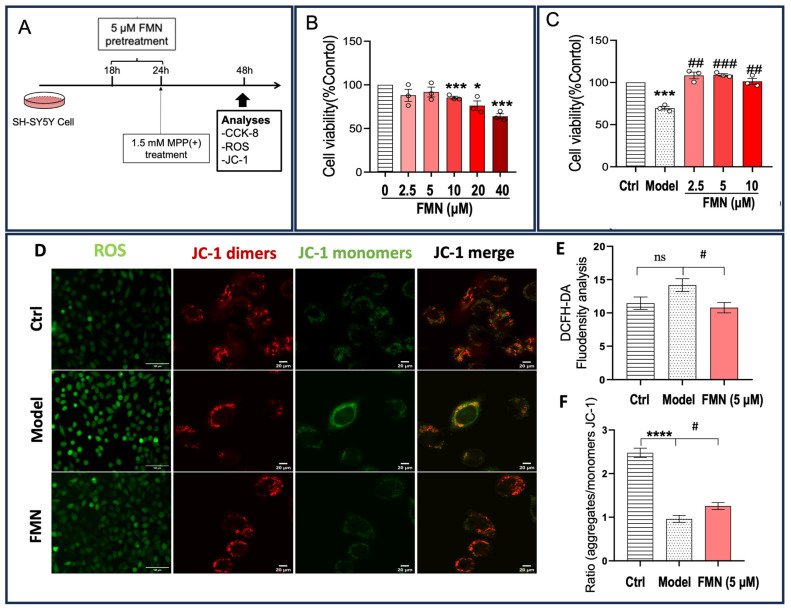
FMN demonstrates neuroprotective effects in MPP(+)-induced SH-SY5Y cells. (**A**) The experimental procedure for treating SH-SY5Y cells with FMN. (**B**) An assessment of FMN toxicity in SH-SY5Y cells (*n* = 3, *N* = 3). (**C**) The effects of varying concentrations of FMN on the viability of SH-SY5Y cells exposed to MPP(+) (*n* = 3; *N* = 3). (**D**) Representative fluorescent images illustrating the effects of FMN (5 μM) on the ROS levels and mitochondrial membrane potential (JC-1) in MPP(+)-exposed SH-SY5Y cells. (**E**) The quantification of ROS fluorescence in SH-SY5Y cells (*n* = 7; *N* = 3). (**F**) The quantification of JC-1 dye in SH-SY5Y cells (*n* = 13; *N* = 3). *N* = the number of independent experiments; *n* = the number of nematodes in each independent experiment. In (**B**), an asterisk (*) indicates significant differences between the FMN and Ctrl groups, while in (**C**–**F**), an asterisk (*) indicates significant differences between the Model and Ctrl groups. A hash mark (#) indicates significant differences between the FMN and Model groups. Ns indicates no significant difference between the Model and Ctrl groups. * *p* < 0.05, *** *p* < 0.001, **** *p* < 0.0001, # *p* < 0.05, ## *p* < 0.01, and ### *p* < 0.001.

To determine the optimal concentration of FMN, SH-SY5Y cells were exposed to varying concentrations of FMN for 6 h and subsequently treated with MPP(+) for 24 h. Cell viability was then assessed using the CCK-8 assay. The results indicate that concentrations of FMN exceeding 10 μM were cytotoxic; in contrast, concentrations of 2.5 μM, 5μM, and 10 μM significantly restored cell viability in MPP(+)-treated SH-SY5Y cells (Figure 3A–C). Based on these findings, 5 µM FMN was selected as the optimal concentration for subsequent experiments as it significantly restored cell viability without inducing cytotoxicity. This concentration was used to assess the effects of FMN on ROS production and mitochondrial membrane potential (MMP) in MPP(+)-treated SH-SY5Y cells.

DCFH-DA is widely used to measure intracellular ROS levels [40]. To assess whether FMN could protect SH-SY5Y cells from MPP(+)-induced ROS generation, intracellular ROS levels were determined using DCFH-DA staining. The stained cells were then observed and quantified using an inverted fluorescent microscope. As shown in Figure 3D,E, MPP(+) exposure significantly increased intracellular ROS levels in SH-SY5Y cells compared to the control group. In contrast, FMN treatment markedly reduced ROS levels in MPP(+)-treated SH-SY5Y cells. These results indicate that FMN could mitigate ROS production in SH-SY5Y cells exposed to MPP(+).

ROS production is closely linked to mitochondrial function [41,42], and the MMP serves as an indicator of mitochondrial functionality. JC-1 is a commonly used dye for measuring the MMP [43,44]. Specifically, JC-1 accumulates in the mitochondrial matrix, forming a polymer that emits red fluorescence at high MMP. Conversely, at low MMP, JC-1 exists as a monomer and emits green fluorescence. In this study, JC-1 was employed to evaluate the protective effects of FMN on mitochondrial function. The results indicate that MMP was significantly reduced in SH-SY5Y cells exposed to MPP(+); however, MMP levels were restored with FMN treatment (Figure 3F). These findings suggest that FMN could restore MMP in SH-SY5Y cells exposed to MPP(+).

### 2.4. The Proposed Mechanism for the Anti-PD Effects of FMN Through the Activation of the Nrf2 Signaling Pathway

**Figure 4:**
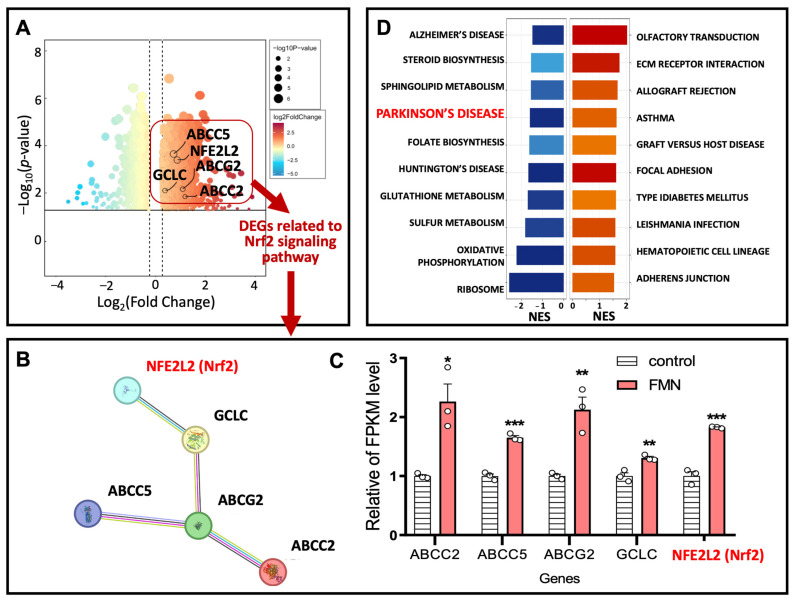
The proposed mechanism for FMN’s anti-PD effects through the activation of the Nrf2 signaling pathway. (**A**) A volcano plot of the DEGs identified from the transcriptome analysis following FMN treatment. The DEGs related to the Nrf2 signaling pathway that were upregulated by FMN are highlighted with a red frame. Transcriptome data were downloaded and analyzed using an online ITCM database (http://itcm.biotcm.net/, analyzed on 6 August 2023). (**B**) A PPI network and (**C**) the relative expression profiles of Nrf2-associated genes among the DEGs identified from the FMN-treated sample transcriptome data. (**D**) A disease-related enrichment analysis of the DEGs derived from the transcriptome data of the FMN-treated samples, with relevance to PD highlighted in red. An asterisk (*) indicates significant differences between the Model and Ctrl groups; * *p* < 0.05, ** *p* < 0.01 and *** *p* < 0.001.

To identify the signaling pathway through which FMN exerts its anti-PD effects, we analyzed transcriptome data following FMN treatment. Our analysis revealed that FMN significantly activated the expression of Nrf2 and Nrf2-related genes, including GCLC, ABCC2, ABCC5, and ABCG2 (Figure 4A–C). Furthermore, the results of the disease enrichment analysis of differentially expressed genes (DGEs) from FMN treatment indicated that FMN may have therapeutic potential for PD (Figure 4D), corroborating our findings.

Nrf2 is recognized as an important transcription factor that mediates oxidative stress, which can be significantly alleviated through the activation of the Nrf2 signaling pathway [45,46]. Moreover, our results demonstrate that FMN significantly reduces oxidative stress in PD models. Therefore, our transcriptome data indicate that FMN may exert its anti-PD effects by activating the Nrf2 signaling pathway.

### 2.5. The Nrf2 Inhibitor Could Negate the Protective Effects of FMN

**Figure 5:**
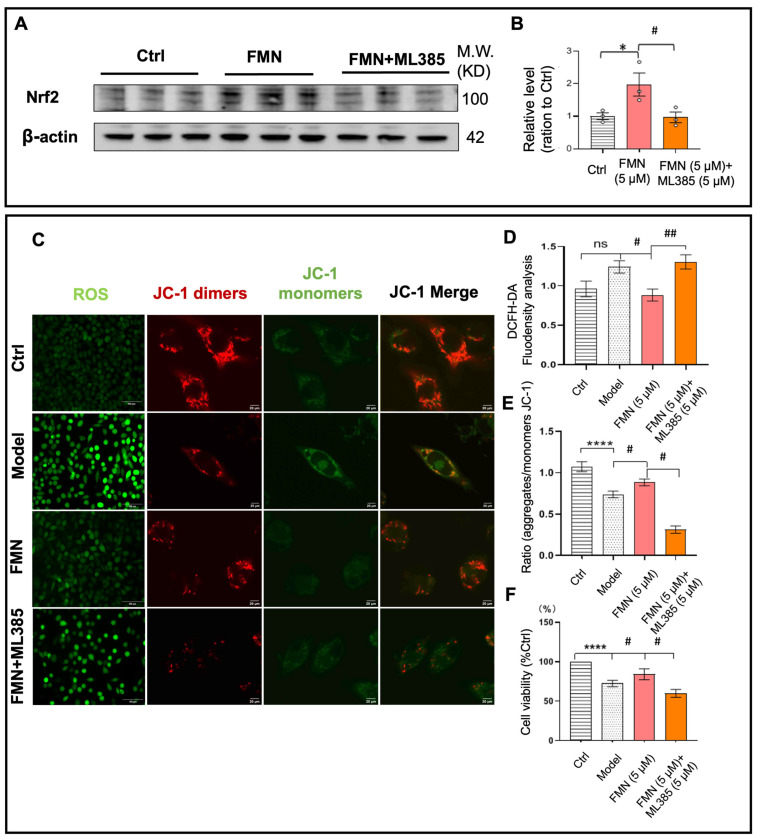
The Nrf2-specific inhibitor (ML385) blocks the beneficial effects of FMN in MPP(+)-treated SH-SY5Y cells. (**A**) A Western blot analysis of nuclear-translocated Nrf2 protein. (**B**) The quantification of nuclear-translocated Nrf2 protein (*n* = 3; *N* = 3). (**C**) Representative fluorescence images of ROS and JC-1 staining in SH-SY5Y cells exposed to MPP(+). (**D**) The quantification of ROS fluorescence in MPP(+)-treated SH-SY5Y cells (*n* = 4; *N* = 3). (**E**) The quantification of JC-1 fluorescence in SH-SY5Y cells exposed to MPP(+) (*n* = 15; *N* = 3). (**F**) The viability of SH-SY5Y cells exposed to MPP(+) (*n* = 4; *N* = 3). *N* represents the number of independent experiments, and *n* represents the number of samples per experiment. In (**B**), an asterisk (*) denotes significant differences between the FMN and Ctrl groups, while in (**D**–**F**), an asterisk (*) denotes significant differences between the Model and Ctrl groups. A hash mark (#) indicates significant differences between the FMN and Model groups or between the FMN+ML385 and FMN groups. Ns indicates no significant difference between the Model and Ctrl groups. * *p* < 0.05, **** *p* < 0.0001, # *p* < 0.05, and ## *p* < 0.01.

Nrf2 can be activated and translocated to the nucleus, where it binds to the promoter ARE in gene promoters, initiating the expression of antioxidant enzymes [47]. ML385, a specific Nrf2 inhibitor, is characterized by its quinoline-based structure. It binds to the Neh1 domain of Nrf2, thereby inhibiting Nrf2’s ability to interact with the ARE in target gene promoters, which reduces the expression of antioxidant enzymes [48,49]. To confirm whether Nrf2 activation contributes to the anti-PD effects of FMN, we used a non-toxic concentration of ML385 to inhibit FMN-induced Nrf2 activation. The Western blot (WB) analysis results reveal that FMN significantly promoted Nrf2 nuclear translocation, whereas ML385 effectively blocked this translocation (Figure 5A,B). These results suggest that ML385 effectively inhibits FMN-induced Nrf2 activation.

Our results demonstrate that FMN significantly reduced ROS production and restored MMP and cell viability in SH-SY5Y cells exposed to MPP(+). Therefore, we further evaluated whether ML385 could block FMN’s beneficial effects on ROS, MMP, and cell viability by inhibiting Nrf2 activation. Our results show that FMN significantly reduced MPP(+)-induced intracellular ROS; however, ML385 effectively blocked this reduction (Figure 5C,D). Similarly, FMN significantly improved MMP and cell viability; however, these effects were notably blocked by ML385 (Figure 5E,F).

These findings demonstrate that FMN’s anti-PD effects are closely associated with Nrf2 activation.

### 2.6. FMN Exhibited Neuroprotective Effects Comparable to the Antioxidant SFN in C. elegans PD Models

**Figure 6:**
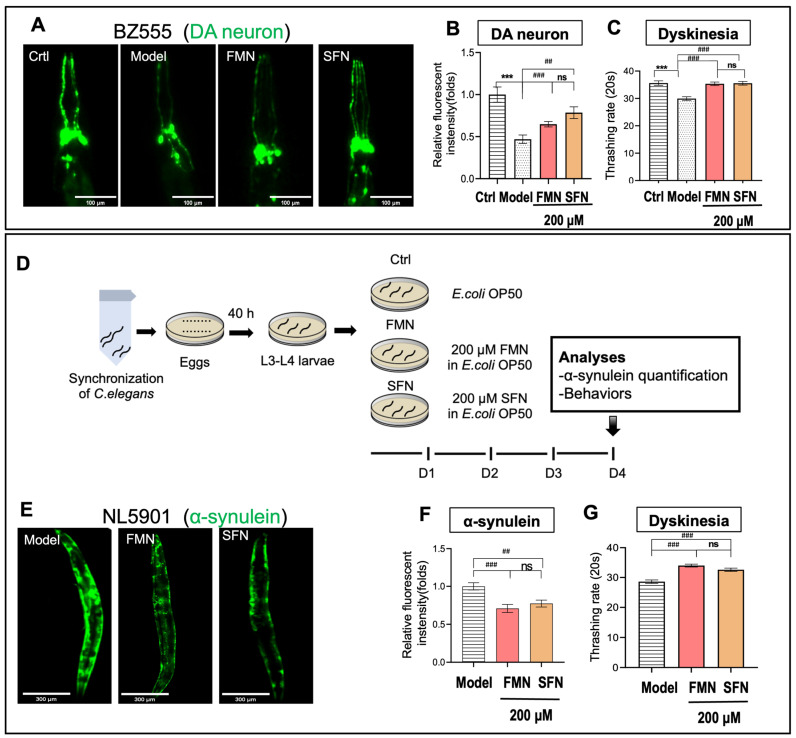
FMN exhibits anti-PD effects comparable to the antioxidant SFN in *C. elegans*. (**A**,**B**) The effects of FMN on the impairment of dopamine neuron damage and motor ability in MPP(+)-induced *C. elegans* BZ555. (**A**) Representative fluorescent images showing the effect on GFP-labeled dopamine neurons (green) in the head region of nematodes. Scale bar = 100 μm. (**B**) The quantification of dopamine neuron fluorescence (*n* = 15; *N* = 3). (**C**) Motor ability measured through a thrashing rate analysis (*n* = 15; *N* = 3). (**D**) The experimental procedure for NL5901 worms: *C. elegans* at L4 were treated with 200 μM FMN or SFN for 4 days at 20 °C, with untreated worms serving as the control (Ctrl). (**F**,**G**) The effects of FMN or SFN on α-synuclein aggregation and motor ability in *C. elegans* NL5901. (**E**) Representative fluorescent images showing the effect of FMN on GFP-labeled α-synuclein (green) in the body wall muscle cells of nematodes. Scale bar = 300 μm. (**F**) The quantification of α-synulein fluorescence. *n* = 15; *N* = 3. (**G**) Motor ability measured through a thrashing rate analysis (*n* = 15: *N* = 3). *N* = the number of independent experiments, *n* = number of nematodes per independent experiment. In (**B**,**C**), an asterisk (*) indicates a significant difference between the FMN and Ctrl groups, while a hash mark (#) indicates a significant difference between any two of the Model, FMN, and SFN groups. In (**F**,**G**), an asterisk (*) denotes a significant difference between the FMN group or SFN group and the Model group. Ns indicates no significant difference between the FMN and SFN groups. *** *p* < 0.001, ## *p* < 0.01, and ### *p* < 0.001.

Sulforaphane (SFN) is a well-known Nrf2 activator [50,51,52], and our results indicate that the anti-PD effects of FMN are linked to the activation of the Nrf2 signaling pathway. To compare the pharmacodynamic effects of FMN and SFN, we evaluated their efficacy in *C. elegans* PD models. In the BZ555 strain *C. elegans* PD model, both FMN and SFN significantly reduced the damage of dopamine neurons and blocked damage being caused to motor ability, with no significant difference between the FMN- and SFN-treated groups (Figure 6A–C). In the NL5901 strain *C. elegans* PD model, both FMN and SFN significantly improved motor performance and inhibited α-synuclein aggregation, with no significant difference between the FMN and SFN treatment groups (Figure 6D–G). These results suggest that FMN shows comparable performance to SFN in improving motor performance and protecting dopamine neurons. The above results demonstrate that FMN is comparable to SFN in protecting dopamine neurons and improving motor dysfunction.

## 3. Discussion

FMN is an isoflavone with significant biological activities. Research shows that FMN exhibits anti-inflammatory and antioxidant effects and may improve various neurological disorders, including Alzheimer’s disease (AD) [53,54,55], ischemic stroke [56,57], anxiety [58], and traumatic brain injury (TBI) [59,60,61]. However, little is known about the effects of FMN on PD. In this study, bioinformatics predictions suggest that FMN may have anti-PD effects. We further validated that FMN inhibits ROS generation, stabilizes MPP(+)-induced damage, protects dopaminergic neurons, and improves locomotor function in both in vivo and in vitro tests. Thus, we confirm, for the first time, that FMN could improve PD.

**Figure 7:**
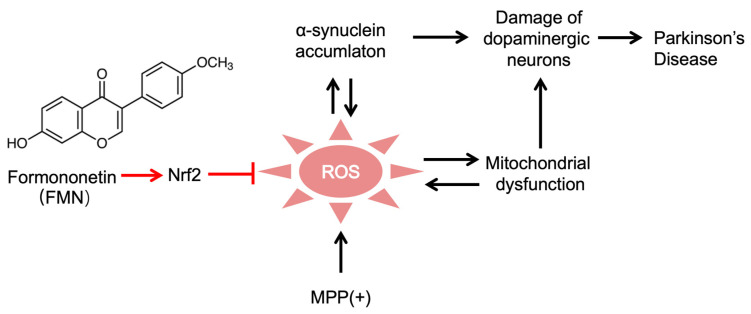
A summary of the effect of FMN on PD. FMN mitigates MPP(+)-induced oxidative stress and protects dopaminergic neurons via Nrf2 activation in a Parkinson’s disease model. MPP(+) increases ROS production, leading to mitochondrial dysfunction and the accumulation of α-synuclein, which further exacerbates neuronal damage. By activating the Nrf2 pathway, FMN helps to mitigate oxidative stress, reduce mitochondrial damage, and protect dopaminergic neurons, potentially delaying or preventing PD progression (created with Biorender, https://app.biorender.com/, accessed on 10 August 2024). The black arrows indicate the known mechanisms by which MPP induces the PD model, while the red arrows and lines represent the mechanism of FMN, which exerts neuroprotective effects by activating Nrf2 to inhibit oxidative stress.

The results of previous studies have shown that the neuroprotective effects of FMN are primarily linked to the reduction in neuroinflammation and oxidative stress [18,25,62]. In AD, FMN activates the PI3K-AKT pathway [53], inhibits the RAGE-NFκB pathway, enhances α-secretase activity, and reduces Tau and Aβ accumulation [54]. In stroke, FMN activates the cAMP-CREB and PI3K-AKT-ERK pathways, promoting neurogenesis and inhibiting apoptosis [57]. In anxiety disorders, FMN promotes neurogenesis and reduces neuroinflammation [58]. In TBI, FMN activates Nrf2, attenuating neuroinflammation and oxidative stress [59,61]. Despite the above findings, it remains unclear whether the beneficial effects of FMN on PD are related to Nrf2 activation. The transcriptomic analysis results obtained in this study suggest that FMN’s anti-PD effects might be linked to Nrf2 signaling activation. Additionally, we found that Nrf2 inhibition blocked FMN’s anti-PD effects. Furthermore, the anti-PD effects of FMN were comparable to those of SFN. Thus, in this study, we demonstrate, for the first time, that FMN’s anti-PD effects are mediated through Nrf2 activation (Figure 7). This study provides new insights into the mechanism by which FMN exerts its neuroprotective effects in PD, specifically through Nrf2 activation, which may offer a valuable foundation for the development of novel therapeutic strategies and supplements for PD.

Although FMN has demonstrated significant neuroprotective effects in preclinical studies, its effects on humans remain unknown. Therefore, further clinical trials are necessary to confirm FMN’s effectiveness and safety.

## 4. Materials and Methods

### 4.1. Bioinformatic Analysis

The targets of FMN were predicted using the following steps: The PubChem database provided the SMILE string of FMN, which was then input into Swiss Target Prediction (validation dataset 2019, http://www.swisstargetprediction.ch/index.php, accessed on 15 March 2024) to screen for potential human targets. The gene names of each protein target were identified using the Uniprot database (version 2024_05, http://www.uniprot.org/, accessed on 15 March 2024). The targets related to PD were identified using the GeneCard database (version 5.22, http://www.genecards.org/, accessed on 15 March 2024), the Therapeutic Targets Database (TTD, last updated in 2024, https://db.idrblab.net/ttd/, accessed on 15 March 2024), and the Online Mendelian Inheritance in Man (OMIM, last updated in 2024, https://www.ncbi.nlm.nih.gov/omim, accessed on 15 March 2024). Only PD-related proteins from Homo sapiens were selected. VENNY (version 2.1, https://bioinfogp.cnb.csic.es/tools/venny/index.html, accessed on 15 March 2024) was used to identify intersecting targets between active ingredients and diseases, after which a PPI network was constructed using String (version 12.0, http://string-db.org/, accessed on 10 May 2024). Next, these targets were entered into the network visualization software Cytoscape (version 3.10.3, http://cytoscape.org/, accessed on 10 May 2024), where five core proteins were selected based on their degree values. Transcriptome data related to FMN were downloaded and analyzed using the Integrated Traditional Chinese Medicine database (ITCM, analyzed on 6 August 2023, http://itcm.biotcm.net) [63].

### 4.2. C. elegans Culture and Treatment

The *C. elegans* strains BZ555 (dat-1p::GFP) and NL5901 (unc-54p::α-synuclein::YFP + unc-119) were obtained from the Caenorhabditis Genetics Center (CGC, University of Minnesota, MN, USA). The nematodes were cultured on nematode growth medium (NGM) agar plates (1.7% agar, 25 mM potassium phosphate, pH 6.0, 50 mM NaCl, 2.5 µg/mL peptone, 5 µg/mL cholesterol, 1 mM MgSO_4_, and 1 mM CaCl_2_) at 20 °C, using *Escherichia coli* (*E. coli*) OP50 (CGC, University of Minnesota, MN, USA) as a food source. The worms were synchronized at L4 prior to treatment.

The BZ555 strain exposed to 4 mM MPP(+) was used as the PD model.

Formononetin (FMN) (Cat. no. F828304, purity of >99.5%) and sulforaphane (SFN) (Cat. no. D818039) were obtained from Macklin Inc., Shanghai, China, with stock solutions prepared in DMSO. Nematodes were treated with different concentrations of FMN or SFN mixed with *E. coli* OP50 on NGM plates for 4 days at 20 °C. For rescue treatment, FMN or SFN was co-administered with MPP(+) to MPP(+)-induced nematodes. The nematodes treated with *E. coli* OP50 alone were used as the control.

### 4.3. Dopaminergic Neurodegeneration Assay in BZ555 Nematodes

The assay of dopaminergic neurodegeneration was performed in MPP(+)-induced BZ555 nematodes as described previously [64]. In brief, the treated nematodes were washed three times with S-basal buffer (50 mM K_2_HPO_4_, 50 mM KH_2_PO_4_, 0.1 mM NaCl) and transferred to 2% agarose pads containing 20 mM levamisole hydrochloride (Cat. no. IL0080, Solarbio, Beijing, China). The GFP fluorescence of the immobilized nematodes was captured using an inverted fluorescence microscope (Revolve, Echo, San Diego, CA, USA) to monitor dopaminergic neuron integrity, and analysis was conducted using ImageJ software (version 1.53e, National Institutes of Health, Bethesda, MD, USA). A minimum of 15 animals were analyzed per group, and all experiments were performed in triplicate.

### 4.4. Thrashing Rate Assay of Nematodes

Following treatment, the nematodes were washed with S-basal buffer to remove *E.coli* OP50 and transferred to 48-well plates containing 200 μL of S-basal buffer, with 5–6 nematodes per well. Fifteen nematodes were randomly selected from each group, and their movement was tracked for 20 s under a stereo microscope (Revolve, Echo, Lake Zurich, IL, USA), with the time being recorded [65]. The number of body bends was counted, and each group was assayed in triplicate.

### 4.5. Measurement of Reactive Oxygen Species (ROS) Levels in C. elegans

The nematodes were collected in a tube and stained for 20 min with S-basal buffer containing 100 µM of a dichlorodihydrofluorescein diacetate (DCFH-DA) (Cat. no. D6470, Solarbio, Beijing, China) fluorescent probe. Subsequently, the nematodes were washed three times with S-basal buffer and transferred to 2% agarose pad slides containing 20 mM levamisole hydrochloride. The nematodes were analyzed using an inverted fluorescence microscope equipped with FITC filters. Fluorescence intensity was quantified using ImageJ software (version 1.53e). A minimum of 15 nematodes were examined in each experiment.

### 4.6. Cell Culture and Treatment

The human neuroblastoma cell line SH-SY5Y (China Cell Resource Confederation, Beijing, China) was cultured in Dulbecco’s Modified Eagle Medium (DMEM, Gibco, New York, NY, USA) supplemented with 10% fetal bovine serum (FBS, Pasching, Austria) and 1% penicillin/streptomycin (PS, Gibco, New York, NY, USA) at 37 °C in a 5% CO_2_ atmosphere.

Cells were seeded at a density of 1 × 10^5^ cells/mL (unless indicated) and cultured for 18 h. The cells were pretreated with FMN (5 μM), ML385 (7.5μM), or SFN (5 μM) for 6 h, followed by treatment with 1.5 mM MPP(+) for 24 h, and the control group received DMEM solution.

### 4.7. Measurement of Cell Viability

Cell viability was assessed using the Cell Counting Kit-8 (CCK-8, CK001, Lablead, Beijing, China), and absorbance values were measured at 450 nm using a microplate reader (Molecular Devices, Sunnyvale, CA, USA). The cells were seeded at a density of 1 × 10^5^ cells/mL in 96-well plates and cultured for 12 h prior to treatment. For the toxicity assay, the cells were exposed to different concentrations of MPP(+) or FMN for 24 h. In co-treatments, the cells were pretreated with 5 μM FMN, 7.5 μM ML385, or 5 μM SFN for 6 h before exposure to 1.5mM MPP(+) alone or in combination with either 5 μM FMN or 5 μM SFN for 24 h.

### 4.8. ROS Assay in Cells

The levels of ROS in each group were assessed using a DCFH-DA fluorescent probe. The cells were cultured and treated in 6-well plates, rinsed with phosphate-buffered saline (PBS) (Cat. no. P854529, Solarbio, Beijing, China), and subsequently incubated in DMEM containing DCFH-DA (10 µM) for 30 min. Following three additional washes with PBS, fluorescence was recorded using an inverted fluorescence microscope (Revolve, Echo, San Diego, CA, USA). Fluorescence intensity was analyzed with Image J software (version 1.53e, National Institutes of Health, Bethesda, MD, USA).

### 4.9. Measurement of Mitochondrial Membrane Potential (MMP) in Cells

The MMP was assessed using the fluorescent probe JC-1 (Cat. no. C2006, Beyotime Biotechnology, Beijing, China). The treated cells were rinsed with PBS and incubated with JC-1 staining solution (5 µg/mL JC-1 in double-distilled water) at 37 °C for 30 min, followed by three washes with PBS. JC-1 monomers and aggregates were detected using an inverted confocal microscope (FV3000, Olympus, Tokyo, Japan) equipped with a 100× oil immersion objective. Fluorescence intensity was analyzed using Image J software (version 1.53e, National Institutes of Health, Bethesda, MD, USA).

### 4.10. Western Blot

Cell samples were collected and lysed in 1×RIPA lysis buffer (Cat. no. P0013C, Beyotime Biotechnology, Beijing, China) containing a protease inhibitor cocktail. The protein concentration was normalized by adding loading buffer (5×) and double-distilled water (ddH_2_O) for protein denaturation. The samples (10 μg per lane) were separated via SDS-PAGE gel electrophoresis and subsequently transferred to Polyvinylidene Fluoride (PVDF) membranes. The membranes were blocked with Tris-buffered saline with 0.1% Tween^®^ 20 Detergent (TBST) buffer containing 5% skimmed milk for 2 h at room temperature. They were then incubated overnight at 4 °C with antibodies against Nrf2 (Cat. no. Ab62352, Abcam, Cambridge, UK) and β-actin (Cat. no. A0101, LABLEAD, Beijing, China) after being washed three times with 1× TBST. Subsequently, the membranes were incubated with secondary antibodies, either goat anti-mouse IgG (Cat. no. Y1106, LABLEAD, Beijing, China) or goat anti-rabbit IgG (Cat. no. Y1055, LABLEAD, Beijing, China), for 1 h at room temperature. Lastly, the membranes were washed with 1× TBST, and protein bands were visualized using enhanced chemiluminescence (ECL) detection reagents.

### 4.11. Statistical Analysis

Each experiment was conducted at least three times, with a minimum of three replicates for cell samples and 15 samples for *C. elegans*, unless otherwise specified. Data are presented as means ± SEMs. Data were analyzed using Student’s *t*-test for comparisons between two groups and Tukey’s multiple comparison test for multiple groups across all experiments. An asterisk (*) denotes a significant difference between the control (Ctrl) and MPP(+)-treated groups, whereas a hash mark (#) indicates a significant difference between the FMN-treated and MPP(+)-treated groups. Statistical significance was defined as * or # for *p* < 0.05; **or ## for *p* < 0.01; and *** or ### for *p* < 0.001. Statistical analysis was performed using GraphPad Prism 8.0 (GraphPad Software Inc., San Diego, CA, USA).

## Data Availability

Transcriptome data related to FMN (ID:54) can be downloaded from the Integrated Traditional Chinese Medicine database (ITCM, http://itcm.biotcm.net/, accessed on on 6 August 2023).

## Supplementary Materials

The following supporting information can be downloaded at https://www.mdpi.com/article/10.3390/molecules29225364/s1, Table S1: Gene–target interactions, pathways, and disease associations related to Parkinson’s disease and FMN; Figure S1: Thrashing rate of BZ555 *C. elegans* treated with different concentrations of MPP(+); Figure S2: Thrashing rates of N2 strain treated with different concentrations of FMN; Figure S3: Effects of FMN and SFN (200 μM) on cell viability, ROS levels, and mitochondrial membrane potential in SH-SY5Y cells. Refs [66,67,68,69,70,71,72,73,74,75,76,77,78,79,80,81,82,83,84,85,86,87,88,89,90] are cited in the Supplementary Materials.
