# Supplementary material for: Formononetin Exerts Neuroprotection in Parkinson’s Disease via the Activation of the Nrf2 Signaling Pathway
Source: Molecules. 2024 Nov 14;29(22):5364. doi: 10.3390/molecules29225364 (PMC11596823; doi:10.3390/molecules29225364)
Supplement: Supplementary file 1 [file molecules-29-05364-s001.zip › molecules-3201588-supplementary.pdf]

Table S1. Gene-Target Interactions, Pathways, and Disease Associations Related to Parkinson's Disease and FMN

Protein Symbol	Full Name	Function	Diseases Associated	Pathways Involved	References
PON1	Paraoxonase 1	Exhibits lactonase and ester hydrolase activity. Hydrolyzes thiolactones and xenobiotics.	Microvascular Complications of Diabetes 5, Frontotemporal Dementia	Fatty acid metabolism, Drug ADME	[1]
MAOB	Monoamine Oxidase B	Catalyzes oxidative deamination of biogenic and xenobiotic amines.	Personality Disorder, Atypical Depressive Disorder	Disulfiram Pathway, Dopaminergic neuron, Oxidation by cytochrome P450	[2]
BACE1	Beta-Secretase 1	Member of the peptidase A1 family of aspartic proteases.	Alzheimer's Disease, Myositis	Alzheimer's disease, miRNA effects, Metabolism of proteins	[3]
EGFR	Epidermal Growth Factor Receptor	Cell surface protein inducing receptor dimerization and tyrosine autophosphorylation.	Inflammatory Skin and Bowel Disease, Lung Cancer	Apoptotic Pathways in Synovial Fibroblasts, Signaling by EGFR in Cancer	[4, 5]
IL2	Interleukin 2	Secreted cytokine important for T and B lymphocyte proliferation.	Breast Cancer, Diphtheria	Apoptotic Pathways in Synovial Fibroblasts, MIF Mediated Glucocorticoid Regulation	[6, 7]
ACHE	Acetylcholinesterase (Yt Blood Group)	Hydrolyzes acetylcholine, terminating signal transmission at neuromuscular	Yt Blood Group Antigen, Sickle Cell Disease	Transmission across Chemical Synapses, Glycerophosp	[8]

		junctions.		holipid biosynthesis	
ABCB1	ATP Binding Cassette Subfamily B Member 1	Functions as a transporter in the blood-brain barrier.	Inflammatory Bowel Disease 13, Colchicine Resistance	Drug ADME, Ponatinib Pathway (Pharmacokinetics/Pharmacodynamics)	[9]
MAOA	Monoamine Oxidase A	Catalyzes the oxidative deamination of amines, including dopamine and serotonin.	Brunner Syndrome, Antisocial Personality Disorder	Neurotransmitter clearance, Oxidation by cytochrome P450	[10, 11]
ESR1	Estrogen Receptor 1	Ligand-activated transcription factor involved in hormone signaling.	Estrogen Resistance, Breast Cancer	ESR-mediated signaling, Constitutive Signaling by Aberrant PI3K in Cancer	[12, 13]
HTR2A	5-Hydroxytryptamine Receptor 2A	Receptor for serotonin involved in neuroactivity.	3-methylglutaconic aciduria 8,Parkinson disease	GPCR downstream signaling, Class A/1 Rhodopsin-like receptors	[14, 15]
TYR	Tyrosinase	Catalyzes the conversion of tyrosine to melanin.	Albinism, Oculocutaneous Type Ia/Ib	Pheomelanin biosynthesis, MITF-M-dependent gene expression	[16, 17]
MIF	Macrophage Migration Inhibitory Factor	Lymphokine involved in immune regulation and inflammation.	Rheumatoid Arthritis, Systemic Juvenile Idiopathic Arthritis	Interleukin-12 family signaling, Innate Immune System	[18, 19]
ADOR A2A	Adenosine A2a Receptor	G-protein coupled receptor implicated in inflammatory diseases and neurodegenerative	Acute Encephalopathy, Basal Ganglia Disease	Signaling by NTRKs, Purinergic signaling	[20, 21]

		disorders.			
ALDH2	Aldehyde Dehydrogenase 2 Family Member	Enzyme involved in alcohol metabolism, key in oxidation of aldehydes.	Alcohol Sensitivity, Amed Syndrome	Ethanol degradation II, Oxidation by cytochrome P450	[22]
TLR9	Toll Like Receptor 9	Key receptor in pathogen recognition and innate immunity activation.	Cervicitis, Measles	Toll Like Receptor 7/8 Cascade, Insulin receptor signaling cascade	[23]
CYP19A1	Cytochrome P450 Family 19 Subfamily A	Monooxygenase involved in drug metabolism and synthesis of cholesterol and steroids.	Aromatase Deficiency, Aromatase Excess Syndrome	Oxidation by cytochrome P450, Metapathway biotransformation Phase I/II	[24, 25]

1. Sorenson, R. C.; Bisgaier, C. L.; Aviram, M.; Hsu, C.; Billecke, S.; La Du, B. N., Human serum Paraoxonase/Arylesterase's retained hydrophobic N-terminal leader sequence associates with HDLs by binding phospholipids : apolipoprotein A-I stabilizes activity. Arterioscler Thromb Vasc Biol 1999, 19, (9), 2214-25.
2. Newton-Vinson, P.; Hubalek, F.; Edmondson, D. E., High-level expression of human liver monoamine oxidase B in Pichia pastoris. Protein Expr Purif 2000, 20, (2), 334-45.
3. Okada, H.; Zhang, W.; Peterhoff, C.; Hwang, J. C.; Nixon, R. A.; Ryu, S. H.; Kim, T. W., Proteomic identification of sorting nexin 6 as a negative regulator of BACE1-mediated APP processing. Faseb j 2010, 24, (8), 2783-94.
4. Campbell, P.; Morton, P. E.; Takeichi, T.; Salam, A.; Roberts, N.; Proudfoot, L. E.; Mellerio, J. E.; Aminu, K.; Wellington, C.; Patil, S. N.; Akiyama, M.; Liu, L.; McMillan, J. R.; Aristodemou, S.; Ishida-Yamamoto, A.; Abdul-Wahab, A.; Petrof, G.; Fong, K.; Harnchoowong, S.; Stone, K. L.; Harper, J. I.; Irwin McLean, W. H.; Simpson, M. A.; Parsons, M.; McGrath, J. A., Epithelial inflammation resulting from an inherited loss-of-function mutation in EGFR. J Invest Dermatol 2014, 134, (10), 2570-2578.
5. Paez, J. G.; Jänne, P. A.; Lee, J. C.; Tracy, S.; Greulich, H.; Gabriel, S.; Herman, P.; Kaye, F. J.; Lindeman, N.; Boggon, T. J.; Naoki, K.; Sasaki, H.; Fujii, Y.; Eck, M. J.; Sellers, W. R.; Johnson, B. E.; Meyerson, M., EGFR mutations in lung cancer: correlation with clinical response to gefitinib therapy. Science 2004, 304, (5676), 1497-500.
6. Mingari, M. C.; Gerosa, F.; Carra, G.; Accolla, R. S.; Moretta, A.; Zubler, R. H.; Waldmann, T. A.; Moretta, L., Human interleukin-2 promotes proliferation of activated B cells via surface receptors similar to those of activated T cells. Nature 1984, 312, (5995), 641-3.

7. Laâbi, Y.; Gras, M. P.; Carbonnel, F.; Brouet, J. C.; Berger, R.; Larsen, C. J.; Tsapis, A., A new gene, BCM, on chromosome 16 is fused to the interleukin 2 gene by a t(4;16)(q26;p13) translocation in a malignant T cell lymphoma. Embo j 1992, 11, (11), 3897-904.
8. Yang, L.; He, H. Y.; Zhang, X. J., Increased expression of intranuclear AChE involved in apoptosis of SK-N-SH cells. Neurosci Res 2002, 42, (4), 261-8.
9. Choi, K. H.; Chen, C. J.; Kriegler, M.; Roninson, I. B., An altered pattern of cross-resistance in multidrug-resistant human cells results from spontaneous mutations in the mdr1 (P-glycoprotein) gene. Cell 1988, 53, (4), 519-29.
10. Son, S. Y.; Ma, J.; Kondou, Y.; Yoshimura, M.; Yamashita, E.; Tsukihara, T., Structure of human monoamine oxidase A at 2.2-A resolution: the control of opening the entry for substrates/inhibitors. Proc Natl Acad Sci U S A 2008, 105, (15), 5739-44.
11. Brunner, H. G.; Nelen, M.; Breakefield, X. O.; Ropers, H. H.; van Oost, B. A., Abnormal behavior associated with a point mutation in the structural gene for monoamine oxidase A. Science 1993, 262, (5133), 578-80.
12. Molli, P. R.; Singh, R. R.; Lee, S. W.; Kumar, R., MTA1-mediated transcriptional repression of BRCA1 tumor suppressor gene. Oncogene 2008, 27, (14), 1971-80.
13. Quaynor, S. D.; Stradtman, E. W., Jr.; Kim, H. G.; Shen, Y.; Chorich, L. P.; Schreihofer, D. A.; Layman, L. C., Delayed puberty and estrogen resistance in a woman with estrogen receptor α variant. N Engl J Med 2013, 369, (2), 164-71.
14. Bartke, T.; Pohl, C.; Pyrowolakis, G.; Jentsch, S., Dual role of BRUCE as an antiapoptotic IAP and a chimeric E2/E3 ubiquitin ligase. Mol Cell 2004, 14, (6), 801-11.
15. Strauss, K. M.; Martins, L. M.; Plun-Favreau, H.; Marx, F. P.; Kautzmann, S.; Berg, D.; Gasser, T.; Wszolek, Z.; Müller, T.; Bornemann, A.; Wolburg, H.; Downward, J.; Riess, O.; Schulz, J. B.; Krüger, R., Loss of function mutations in the gene encoding Omi/HtrA2 in Parkinson's disease. Hum Mol Genet 2005, 14, (15), 2099-111.
16. Lai, X.; Wichers, H. J.; Soler-Lopez, M.; Dijkstra, B. W., Structure of Human Tyrosinase Related Protein 1 Reveals a Binuclear Zinc Active Site Important for Melanogenesis. Angew Chem Int Ed Engl 2017, 56, (33), 9812-9815.
17. Tsai, C. H.; Tsai, F. J.; Wu, J. Y.; Lin, S. P.; Chang, J. G.; Yang, C. F.; Lee, C. C., Insertion/deletion mutations of type I oculocutaneous albinism in chinese patients from Taiwan. Hum Mutat 1999, 14, (6), 542.
18. Tan, T. H.; Edgerton, S. A.; Kumari, R.; McAlister, M. S.; Roe, S. M.; Nagl, S.; Pearl, L. H.; Selkirk, M. E.; Bianco, A. E.; Totty, N. F.; Engwerda, C.; Gray, C. A.; Meyer, D. J., Macrophage migration inhibitory factor of the parasitic nematode Trichinella spiralis. Biochem J 2001, 357, (Pt 2), 373-83.
19. Donn, R. P.; Shelley, E.; Ollier, W. E.; Thomson, W., A novel 5'-flanking region polymorphism of macrophage migration inhibitory factor is associated with systemic-onset juvenile idiopathic arthritis. Arthritis Rheum 2001, 44, (8), 1782-5.
20. Franco, R.; Cordomí, A.; Llinas Del Torrent, C.; Lillo, A.; Serrano-Marín, J.; Navarro, G.; Pardo, L., Structure and function of adenosine receptor heteromers. Cell Mol Life Sci 2021, 78, (8), 3957-3968.
21. Pedata, F.; Pugliese, A. M.; Coppi, E.; Dettori, I.; Maraula, G.; Cellai, L.; Melani, A., Adenosine A2A receptors modulate acute injury and neuroinflammation in brain ischemia. Mediators Inflamm 2014, 2014, 805198.

22. Oka, Y.; Hamada, M.; Nakazawa, Y.; Muramatsu, H.; Okuno, Y.; Higasa, K.; Shimada, M.; Takeshima, H.; Hanada, K.; Hirano, T.; Kawakita, T.; Sakaguchi, H.; Ichimura, T.; Ozono, S.; Yuge, K.; Watanabe, Y.; Kotani, Y.; Yamane, M.; Kasugai, Y.; Tanaka, M.; Suganami, T.; Nakada, S.; Mitsutake, N.; Hara, Y.; Kato, K.; Mizuno, S.; Miyake, N.; Kawai, Y.; Tokunaga, K.; Nagasaki, M.; Kito, S.; Isoyama, K.; Onodera, M.; Kaneko, H.; Matsumoto, N.; Matsuda, F.; Matsuo, K.; Takahashi, Y.; Mashimo, T.; Kojima, S.; Ogi, T., Digenic mutations in ALDH2 and ADH5 impair formaldehyde clearance and cause a multisystem disorder, AMeD syndrome. Sci Adv 2020, 6, (51).
23. Takeshita, F.; Leifer, C. A.; Gursel, I.; Ishii, K. J.; Takeshita, S.; Gursel, M.; Klinman, D. M., Cutting edge: Role of Toll-like receptor 9 in CpG DNA-induced activation of human cells. J Immunol 2001, 167, (7), 3555-8.
24. Sohl, C. D.; Guengerich, F. P., Kinetic analysis of the three-step steroid aromatase reaction of human cytochrome P450 19A1. J Biol Chem 2010, 285, (23), 17734-43.
25. Bouchoucha, N.; Samara-Boustani, D.; Pandey, A. V.; Bony-Trifunovic, H.; Hofer, G.; Aigrain, Y.; Polak, M.; Flück, C. E., Characterization of a novel CYP19A1 (aromatase) R192H mutation causing virilization of a 46,XX newborn, undervirilization of the 46,XY brother, but no virilization of the mother during pregnancies. Mol Cell Endocrinol 2014, 390, (1-2), 8-17.

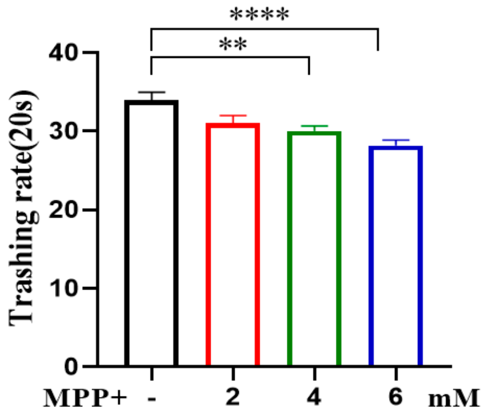

Figure S1. Trashing rate of BZ555 *C. elegans* treated with different concentrations of MPP(+). ** $p < 0.01$. **** $p < 0.0001$.

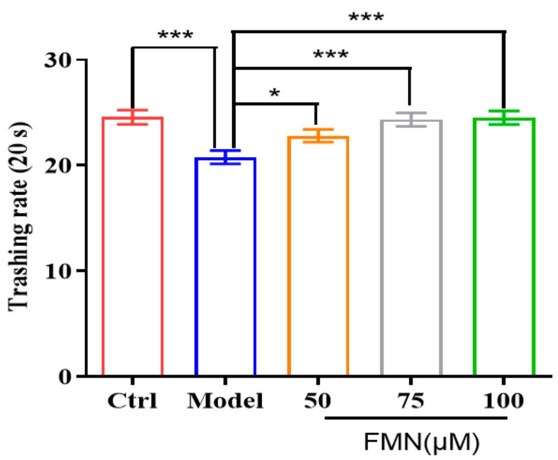

Figure S2. Trashing rates of N2 strain treated with different concentrations of FMN. The number of body bends was recorded over a specified time period (e.g., 20 seconds) for control, model, and FMN-treated groups at concentrations of 10, 50, and 100 µM. Data are presented as mean ± SEM. Asterisks indicate statistically significant differences: ***$P < 0.001$, *$P < 0.05$.

(A) (B)

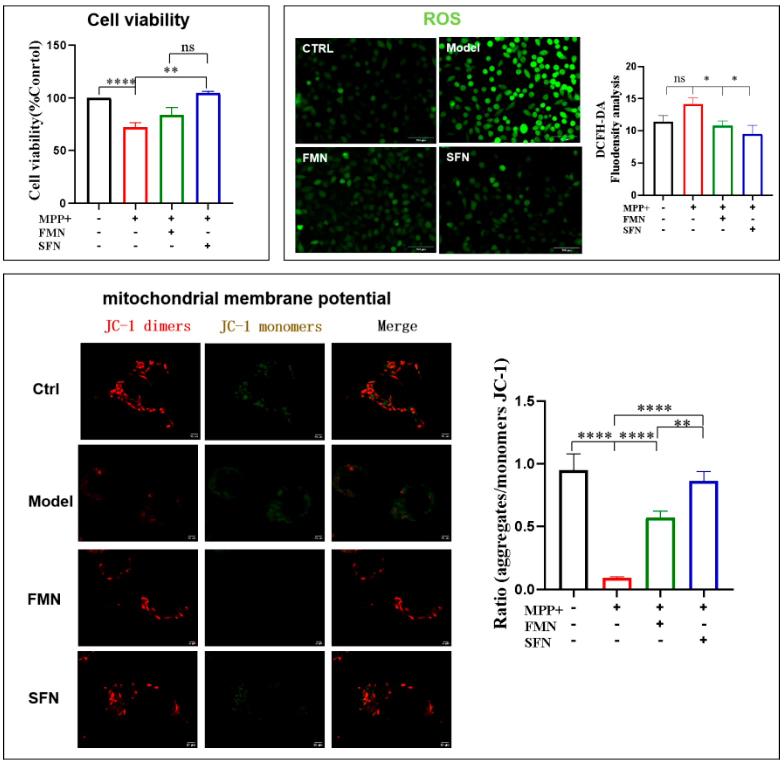

(C)

Figure S3. Effects of FMN and SFN (200 µM) on cell viability, ROS levels, and mitochondrial membrane potential in SH-SY5Y cells. SH-SY5Y cells were treated with 200 µM FMN or SFN for 24 hours and then evaluated. (A) Cell viability was measured using the MTT assay, showing that both FMN and SFN significantly increased cell viability. (B) ROS levels were assessed using the DCFH-DA fluorescent probe, with FMN and SFN treatments significantly reducing ROS levels compared to the model group. (C) Mitochondrial membrane potential was analyzed using the JC-1 assay, indicating that both treatments effectively mitigated the loss of mitochondrial membrane potential. Data are presented as mean ± SEM (n=3), *$P < 0.05$, **$P < 0.01$. **** $p < 0.0001$.
